# Hormonal Link to Autoimmune Allergies

**DOI:** 10.5402/2012/910437

**Published:** 2012-08-22

**Authors:** Shilpa Shah

**Affiliations:** Division of Science, University of Mumbai, Mumbai 400032, India

## Abstract

IgE recognition of autoantigens might augment allergic inflammation in the absence of exogenous allergen exposure. Among allergy and autoimmunity, there is disproportionate representation of males before puberty and females after puberty, suggesting a role for sex hormones. Hormone allergy is an allergic reaction where the offending allergens are one's own hormones. It is an immune reaction to the hormones, which can interfere with the normal function of the hormones. It can occur perimenstrually in women along with the variation in menstrual cycle. The perimenstrual allergies are about the cyclic abundance of the hormone causing a cyclic expression of allergic symptoms. The inflammatory mechanisms of allergic reactions to hormone allergens, which are intrinsic to the body, are the same as the mechanisms of allergic reactions to external allergens.

## 1. Introduction

Allergy is a hypersensitivity disease based on body's immune recognition of external allergens when they are inhaled, ingested, or contacted. Exposure of allergic individuals to external allergens can lead to immediate type inflammation caused by degranulation of mast cells via IgE-allergen immune complexes and the release of inflammatory mediators, proteases, and proinflammatory cytokines. However, allergic inflammation is reported to occur and persist in the absence of exposure to exogenous allergens and might paradoxically resemble a Th1-mediated chronic inflammatory reaction. There is evidence supporting the view that autoimmune mechanisms might contribute to these processes. IgE recognition of autoantigens might augment allergic inflammation in the absence of exogenous allergen exposure. Moreover, autoantigens that activate Th1-immune responses could contribute to chronic inflammation in allergy, thus linking allergy to autoimmunity [1].

Among allergy and autoimmunity, there is disproportionate representation of males before puberty and females after puberty, suggesting a role for sex hormones. After puberty, female allergy sufferings report more severe symptoms and a greater number of emergency room and hospital admissions than males [2–4]. Further majority of people living with autoimmune disorders are women as well. In fact, autoimmune diseases are among the leading causes of morbidity in females. An estimated 75 percent of those living with autoimmune diseases are females [5–7].

This gender dimorphism in the immune function of females could be due to sex hormones. In addition to their effects on sexual differentiation and reproduction, sex hormones influence the immune system. This theory is supported by observations that the female immune response changes throughout the menstrual cycle. One study examining skin prick testing (SPT) in women with aeroallergens reported significantly increased wheel-and-flare responses on days 12–16 of the menstrual cycle which correspond to peak estrogen levels [8]. Kirmaz et al. compared allergen SPT with serum hormone levels in 42 women with seasonal allergies. They found that oestradiol and luteinizing hormones were correlated with SPT response at midcycle [9]. The menstrual phase has also been shown to influence nasal reactivity, as the period of peak estrogen is correlated with the nasal mucosa becoming hyperreactive to histamine [10]. The symptoms associated with some autoimmune diseases change with natural changes in estrogen and progesterone such as those that occur during the menstrual cycle, pregnancy, and at menopause [5–7]. Annals of Allergy Asthma and Immunology had published a study by Haggerty et al. [11] in 2003 titled “The impact of estrogen and progesterone on asthma.” This study was based on Medline articles published during the year 1966 to 2001, about asthma, pulmonary function, menarche, menopause, estrogen, progesterone, hormone replacement therapy, oral contraceptives, and menstrual cycle. The study concluded that “Estrogen and progesterone modify airway responsiveness.” Further, a recently published study has reported that estrogen stimulates Th2 cytokine production and regulates the compartmentalisation of eosinophils during allergen challenge in a mouse model of asthma [12]. Thus, female sex hormones support a more robust antibody response to allergens and autoantigens [13].

In addition to the evidences indicating a role for female sex hormones in allergy and autoimmunity [14, 15], there are also reports indicating that allergies, autoimmune diseases, fibromyalgia, chronic fatigue syndrome, and hypothyroidism are significantly more common in women with disease conditions due to hormone imbalance like endometriosis than in women in the general population [16]. Studies of women indicate associations of asthma with natural hormonal status like puberty [17], menstrual cycle [16], pregnancy [18], and menopause [19].

Allergy and autoimmune diseases are increasing around the world. Diagnosing such problems and helping those affected to have an improved quality of life is thus a growing concern of physicians worldwide. Although there is information on the relationship of hormones, allergy, and autoimmune diseases, this relationship is, however, poorly understood. 

## 2. Hormone Allergy and Hormone Allergens

It may not be a familiar idea that a physiological body constituent can be an allergen. There are other examples of type I hypersensitivity induced by an endogenous protein or chemical. IgE responses to endogenous proteins can be triggered by any condition that causes necrosis, because intracellular proteins are released that do not otherwise have access to IgE or any other surface-bound antigen receptor. For example,Polacek et al. [20] have reported a characteristic finding, that there was a rapid and dramatic increase of IgE in the serum of all patients with severe burn injury. Some of them showed up to 20-fold increase. IgE was also found in considerable amounts in blister fluid and pleural effusions. In some patients, raised IgE values were the only indicator of an immediate allergic state. Thus simple trauma to the skin is not a sufficient explanation for the elevated serum IgE levels in atopic dermatitis and generalized neurodermatitis. Some other mechanisms might be involved.

Hormone allergy is an allergic reaction where the offending allergens are one's own hormones. It is an immune reaction to the hormones, which can interfere with the normal function of the hormones. It can occur perimenstrually in women along with the variation in menstrual cycle. The perimenstrual allergies are about the cyclic abundance of the hormone causing a cyclic expression of allergic symptoms. All hormones can act as allergens. Different derivatives of estradiol and progesterone bound to serum proteins such as bovine serum albumin (BSA), for example, *β*-estradiol-6-carboxymethyl-BSA, *β*-estradiol-6-carboxymethyl-BSA, progesterone-11-*α*-BSA, progesterone-7-BSA, progesterone-3-carboxymethyl-BSA, and so forth are commercially available. These protein-conjugated hormones are injected into animals to prepare monoclonal and polyclonal antibodies against them. The ease with which these antibodies are formed is strong evidence for the immunogenicity of estrogen and progesterone after their binding to carrier proteins [21].

## 3. Inflammatory Mechanisms of Hormone Allergy

The inflammatory mechanisms of allergic reactions to hormone allergens, which are intrinsic to the body, are the same as the mechanisms of allergic reactions to external allergens. The difference is in the magnitude and manifestation of discomforts. As the body is not constantly being exposed to external allergens, the discomforts in response to allergen exposure are not continuous. Hormones being within the body can induce allergies that result in chronic ailments, although the expression of symptoms can vary with the day of the menstrual cycle in females depending on the hormone load. 

Estrogens may support allergic reactivity in females by acting via the estrogen alpha receptor on mast cells, which might explain the peaking allergic reactions in females around menstruation and pregnancy, under oral contraceptives and hormone replacement therapy. Interestingly, even exogenous or xenoestrogens imitate or support the action of the estrogens. Environmental estrogens like the pesticide lindane accumulate in the environment and may affect the development of the female reproductive system by acting as endocrine disrupters [22]. For allergy, it is important to note that xenoestrogens interact equally well with the estrogen alpha and beta receptors and, thus, may either directly or in conjunction with an allergic reaction support the release of histamine [23, 24]. Additionally, it may be noted in this context that on account of structural homology, phyto-estrogens might also interfere with estrogen receptors and consequently influence mast cell mediator release [25, 26]. The expression of progesterone receptors is upregulated by estrogen through estrogen receptors [27].

## 4. Hormone Allergy Reactions

It is possible that several immune reactions are at work in the process of hormone allergy. One of these possibilities may be that estrogen, progesterone, and their metabolites may act as antigens after binding to different proteins, promoting Th2 cell development, and thereby regulating the synthesis of IgE or other antibodies. The binding of these antibodies to mast cells with their corresponding antigens (hormones or metabolites) induces mast cell or basophile degranulation. This reaction leads to histamine release, Th2 cytokine, and leukotriene secretion, resulting in Type I allergic disease. A second possibility is that after hormones bind to blood proteins, different lymphocytes will react to this complex and induce lymphocyte proliferation and cytokine production, resulting in Type IV allergic reaction or delayed-type hypersensitivity with Th1 predominance [28].

## 5. Pathophysiology of Hormone Allergy

An enzyme known as cytochrome P-450 1B1 converts 17 *α*-estradiol to 4-HE (hydroxyl estradiol) [29, 30]. Further, numerous enzymes can change 4-HE into compounds called 3,4-semiquinones and 3,4-quinones. Though estrogen, progesterone, and other hormones are present in the body in tiny quantities, hormones are also synthesized directly in cells in target tissues, far exceeding the amount of the hormone in blood, for example, adipose tissues and adrenal tissues. As the tissue itself makes hormone, there is enough present to make high levels of hormone metabolism leading to protein and DNA adducts. While adducts between cellular proteins and hormone metabolites may induce antihormone and anti-tissue antibodies, the DNA-hormone adducts may trigger DNA damage. Hormone adducts are formed by intercalation of steroid hormones between the stacked bases in the DNA, for example, estrogen-DNA adducts [31, 32].

## 6. The Manifestations of Hormone Allergy

The manifestations of hormone allergy are as follows:

- premenstrual syndrome,
- premenstrual asthma,
- menstrual migraine,
- weight problems,
- loss of short term memory,
- fatigue,
- skin problems,
- mood swings,
- diminished sex drive,
- anxiety and panic attacks,
- fibromyalgia,
- interstitial cystitis,
- arthritis,
- chronic fatigue syndrome,
- infertility.

## 7. Diagnosing Hormone Allergy

The steps to diagnose hormone allergy are as follows.

**History Taking**

For the female patients it is very important to ask about the year of menarche, and the menstrual history including days of cycle, regular or irregular menses, type of flow, and associated discomforts. The younger they are when they show the first symptoms, the more pronounced could be the problem of hormone allergy.

**Inventory of the System**

Weight problems, loss of short-term memory, fatigue, skin problems, mood swings, diminished sex drive, anxiety and panic attacks, premenstrual syndrome, premenstrual asthma, menstrual migraine, fibromyalgia, interstitial cystitis, arthritis, chronic fatigue syndrome, and infertility all could possibly be an indication of hormone allergy.

Whenever a patient presents with complaints about chronic symptoms although all laboratory tests and other investigations appear to be within the reported normal limits, evaluation for hormone allergies should be performed before dismissal.

**Skin Testing**

Skin test for diagnosing allergy to hormone can be carried out in the same fashion as the routine skin allergy test with other external allergens, for example, inhalants, ingestants, or contactants. Skin prick test or intradermal test or patch test can be carried out using natural bioidentical hormone in question along with a positive (histamine) control and a negative (saline) control.

**Anti-Hormone Specific Antibodies**

Patient's serum can be screened for presence of antibodies specific to the hormones using enzyme linked immunosorbent assay technique.

## 8. Treatment for Hormone Allergy

**Immunotherapy for Hormone Allergy**

For hormone immunotherapy bioidentical hormones obtained from natural sources are diluted with phosphate buffered saline to get solutions of different concentrations. The route of administration may have a strong influence on the efficacy of the treatment since the distribution and concentration of the hormone at the tissue level varies considerably. The sublingual route is preferred over the subcutaneous route for convenience of the patient and better patient compliance. Sublingual administration of hormone dilutions results in rapid absorption of the hormone via the blood vessels under the tongue, and patient gets relief from the troubling symptoms of hormone allergy including asthma, rhinitis, hives, and pain. As immunotherapy with hormone dilutions acts to improve immunoregulation, on continuation of treatment over longer period of time the patient undergoes remission or a complete cure of the disease and sufferings due to hormone allergy [33].
